# A Novel Low-Cost Instrumentation System for Measuring the Water Content and Apparent Electrical Conductivity of Soils

**DOI:** 10.3390/s151025546

**Published:** 2015-10-05

**Authors:** Alan Kardek Rêgo Segundo, José Helvecio Martins, Paulo Marcos de Barros Monteiro, Rubens Alves de Oliveira, Gustavo Medeiros Freitas

**Affiliations:** 1 Department of Control and Automation Engineering and Fundamental Techniques, School of Mines, Federal University of Ouro Preto (UFOP)—campus Morro do Cruzeiro, sn, 35400-000 Ouro Preto, MG, Brazil; E-Mail: pmemop@gmail.com; 2 Department of Agricultural Engineering, Federal University of Viçosa (UFV)—Avenida P.H. Rolfs, sn, 36570-900 Viçosa, MG, Brazil; E-Mails: j.helvecio.martins@gmail.com (J.H.M.); rubensufv1961@gmail.com (R.A.O.); 3 Vale Institute of Technology (ITV)—Avenida Juscelino Kubitschek, 31, Bauxita, 35400-000 Ouro Preto, MG, Brazil; E-Mail: gustavo.medeiros.freitas@itv.org

**Keywords:** dielectric constant, electrical conductivity, self-balancing bridge, microcontroller, embedded system

## Abstract

The scarcity of drinking water affects various regions of the planet. Although climate change is responsible for the water availability, humanity plays an important role in preserving this precious natural resource. In case of negligence, the likely trend is to increase the demand and the depletion of water resources due to the increasing world population. This paper addresses the development, design and construction of a low cost system for measuring soil volumetric water content (θ), electrical conductivity (σ) and temperature (*T*), in order to optimize the use of water, energy and fertilizer in food production. Different from the existing measurement instruments commonly deployed in these applications, the proposed system uses an auto-balancing bridge circuit as measurement method. The proposed models to estimate θ and σ and correct them in function of *T* are compared to the ones reported in literature. The final prototype corresponds to a simple circuit connected to a pair of electrode probes, and presents high accuracy, high signal to noise ratio, fast response, and immunity to stray capacitance. The instrument calibration is based on salt solutions with known dielectric constant and electrical conductivity as reference. Experiments measuring clay and sandy soils demonstrate the satisfactory performance of the instrument.

## 1. Introduction

Currently the lack of drinking water has become one of the major problems faced by humanity. According to UNICEF, less than half of all people in the world have access to this resource. The most water-consuming activities are related to agricultural irrigation, which has to face the challenges of increasing production, while decreasing the water consumption and the environmental impacts. Because of that, it is crucial to develop and deploy technologies to allow sustainable agriculture [1,2,3].

The measurement of soil electrical properties is an alternative to estimate some of its physicochemical properties, which can aid in the management of irrigation and fertilization, making it suitable for cultivation. The dielectric constant (ε) of soil, for example, highly corralates with soil volumetric water content (θ). Therefore, technologies used to measure this parameter in real time can optimize the timing of irrigation [4]. On other hand, the conductivity (σ) of soil can be used to estimate the degree of soil salinity [5]. Furthermore, each type of crop presents optimal productivity at a given level of soil salinity [4].

The irrigation management based on the measurement of water content θ in real time allows one to restrict the amount of water applied, making its use more efficient [6,7,8,9]. Volumetric soil water content is generally regarded as an easier quantity to use than gravimetric content, particularly in irrigation scheduling and calculations of available soil water content. In addition, monitoring the conductivity σ can indicate when the soil needs correction. This parameter has long been used in the construction of spatial variability maps, which allows one to divide production areas into different management zones [5,10]. Thus, sustainable agriculture can be developed ensuring sufficient resources for future generations [4].

There are some types of sensors on the market to estimate θ and σ. However, in general, these devices are expensive, which may discourage producers from using this technology, particularly when it is necessary to import the equipment. Some researchers have proposed the development of systems for monitoring the electrical parameters of the soil [11,12,13,14,15,16,17,18,19,20].

The low-cost system developed in this study monitors water content θ, conductivity σ and temperature (*T*), and could be used for irrigation and fertilization control systems, providing real time measurements. Given the importance of water conservation, it is important to offer the producer various technological options to solve the problem and especially divulge the need to use instrumentation in irrigation systems.

The complete system for irrigation control is composed of: (i) a sensor to measure the soil θ, σ and *T*; (ii) and an automatic calibration system for θ in function of ε, for a specific soil. This paper describes the proposed instrumentation system for irrigation, consisting of a functional prototype [21], which will be deployed in field experiments for validation.

## 2. Experimental Setup

### 2.1. Probes for Measuring θ, σ and T

Patents US2013073097 and US3882383 describe a probe capable of detecting water content in the soil [22,23]. However, the system is based only on conductivity measurements, and does not consider the dielectric constant of the soil. Probes designed to estimate the soil water content based on conductivity usually do not have good precision, because the results also depend on the soil salinity. Patent US5445178 proposes a sensor for this purpose based only on the dielectric conductivity [24], but it does not measure the soil electrical conductivity.

**Figure 1:**
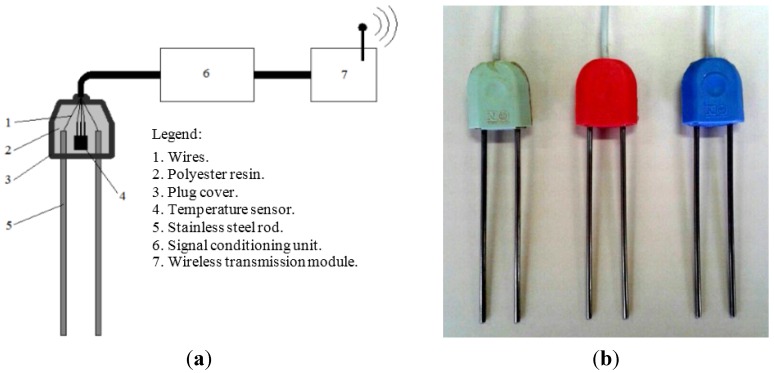
(**a**) Probe for measuring water content and apparent electrical conductivity of soil; (**b**) Probes for measuring temperature, electrical conductivity and relative dielectric constant.

In this work, low-cost probes to measure θ and σ were developed based on measurement of the electrical impedance of the soil material located between two parallel stainless steel rods. In order to construct the probes, stainless steel rods (length = 130 mm, diameter = 3 mm), liquid polyester resin, a semiconductor temperature sensor (LM35), five-way cable and covers for electrical outlets plugs were used, according to the scheme shown in Figure 1a. The cover for electrical outlets plugs served as a housing and to fix the rods and the temperature sensor using polyester resin. Figure 1b illustrates the probes implementation.

### 2.2. Theory and Signal Conditioning Unit

According to Ohm’s law in complex notation, the impedance (**Z**) corresponds to the ratio between the voltage (**V**) and the current (**I**) phasors. This concept assumes that the electrical properties of materials/circuits are time-invariant. The impedance establishes the relationship between module and phase of current and voltage signals in a dipole.

In some cases, it can be more convenient to use the admittance (**Y**)—inverse of impedance, for the analysis of electrical circuits. Equations (1) and (2) represent the impedance and admittance, respectively:

(1) Z=R+jX

(2) Y=G+jB

where *j^2^* = −1, *R* is the resistance and *X* the reactance in Ohms; *G* is the conductance and *B* the susceptance in Siemens. The real part of Equations (1) and (2) is related to the losses by Joule effect. The imaginary part is the ability to exchange energy.

The complex relative permittivity (**ε**) is often used to characterize the electrical properties of materials. This parameter is related to the absolute complex permittivity (**ε_a_**) according to Equation (3):

(3) $${\text{ε}}_{\text{a}}=\varepsilon_{0}\;\text{ε}$$

where ε_0_ is the permittivity of free space (=8.85 pF·m^−1^).

The fluid parameter **ε** presents dielectric relaxation, where the real part decreases with increasing frequency. This phenomenon occurs in the GHz-range. In this work, the frequency range of the measurements is limited to 5 MHz. Therefore, relaxation mechanisms can be neglected, and the complex relative permittivity can be represented by Equation (4):

(4) $$\text{ε}\text{ = ε}-j\frac{\text{σ}}{{\text{ωε}}_{\text{0}}}$$

where ε is the relative dielectric constant (dimensionless), σ the electric conductivity (S·m^−1^) and ω the angular velocity (rad·s^−1^). The imaginary part is the dielectric losses factor.

The impedance measurements of solid, liquid or gaseous substances are carried by means of a probe. Equation (5) lists the electrical properties of the substance around the electrodes, and the probe admittance:

(5) $$\text{Y}=j\text{ω}k_{g}{\text{ε}}_{0}\text{ε}$$

where *k_g_* is the geometric constant of the probe, $G=k_{g}\text{σ}$ and $B=k_{g}{\text{ωε}}_{0}\text{ε}$.

As the impedance is a complex variable, it is necessary to determine two parameters to define it: module and phase, or real and imaginary part of voltage or current. The standard method of impedance measurement consists on applying a pure sinusoidal voltage at a single frequency to the sensor electrodes and measure the phase shift and amplitude, or the real and imaginary parts, of the resulting current using either analog circuit or analog-to-digital conversion and signal processing algorithm to analyze the response [25]. In this context, different measurement techniques have being proposed based on the frequency range, required accuracy, measurement range and complexity of the system [26,27,28].

Measuring voltage and current above 100 MHz are usually difficult, and generally are not directly applicable to high-frequency devices (3–30 MHz), as in the case of the patent US5479104 [29] and US5418466 [30], which work at frequencies up to 100 MHz and 150 MHz, respectively. In this case, the determination of impedance is usually derived from the measurement of wave reflection and transmission together with distributed circuit concepts, such as Theta Probe [31], HidroSense [32], TRIME tube access probe [33]. For this purpose, it is common to employ network analyzers and time domain reflectometers (TDRs), which have higher costs when compared to low frequency measurement systems [27,34].

At lower frequencies (up to MHz range) impedance is determined by current-voltage (or I-V), bridge or resonant methods. The I-V method is quite simple, but has low accuracy. The bridge method has high accuracy, but due to the need to balance the bridge (need of balancing), it is not suitable for fast, repeated and continuous measurements. The resonance method exhibits good accuracy, but also requires adjustment of resonance, which results in the same problem of the bridge method. Moreover, all the above methods are sensitive to stray capacitances to ground, which are normally present in probes due to connecting cables or other grounded metallic parts of a probe, for example. These stray capacitances can impair the measurement impedance, which makes it necessary to use more complex circuitry to eliminate their effect [25].

Sensor 5TE (Decagon Devices Inc., Pullman, WA, USA), for example, provides a 70 MHz wave to the prongs and the bult in microprocessor measures the stored charge, which is proportional to soil ε [13]. This technique requires a third rod to measure ε and σ. Furthermore, this technique does not eliminate the effect of stray capacitances, and requires hardware capable to operate in high frequencies.

**Figure 2:**
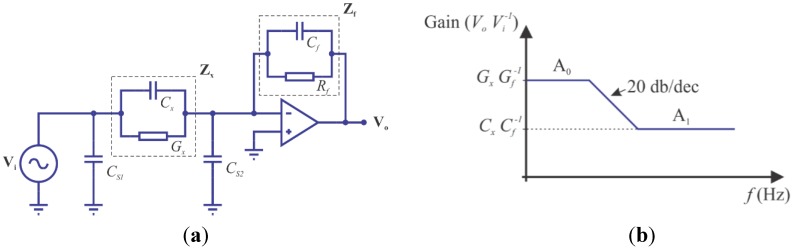
(**a**) Auto-balancing bridge circuit and frequency domanin response (**b**).

Attempting to solve some of these drawbacks, we adopt a variation of the fourth method for measuring impedance, called auto-balancing bridge or self-balancing bridge. This method has a high accuracy, fast response and simple circuit. The measurement is improved with the inclusion of an operational amplifier with high input and low output impedances (Figure 2). This setting is also known as trans-impedance amplifier or current-voltage converter. It has high signal to noise ratio and stray capacitance immunity, capable of measuring small impedances between the electrodes even in the presence of large stray capacitances to ground [25,35].

A measurement system for θ and σ using this measurement method, and neither a low cost embedded system that performs the conditioning and signal processing could not be found in the literature. This circuit allows estimating σ at low frequency (kHz) and ε at high frequency (MHz), requiring only one pair of electrodes in the probe.

The basic auto-balancing bridge circuit diagram is represented in Figure 2a, where **V**_i_ is the input voltage, **Z**_x_ is the unknown impedance, **Z_f_** is the impedance feedback circuit and **V**_o_ is the output voltage. The potential of the operational amplifier non-inverting port is connected to ground potential, which is also known as a virtual ground. Thus, the currents passing through **Z**_f_ and **Z**_x_ are balanced through the operational amplifier action, and the current through the unknown impedance is proportional to the operational amplifier output voltage.

In fact, there are stray capacitances *C_s1_* and *C_s2_*, caused by cables used to connect the sensor to the measuring circuit according to Figure 2b. These capacitances do not affect the measurements since *C_s1_* is drained directly by the voltage source and *C_s2_* is virtually grounded by the operational amplifier. This is a major advantage of auto-balancing bridge circuit [25]. The gain of the circuit shown in Figure 2b is given by Equation (6):

(6) $$\frac{{\text{V}}_{\text{o}}}{{\text{V}}_{\text{i}}}=-\frac{{\text{Z}}_{\text{f}}}{{\text{Z}}_{\text{x}}}=-\frac{{\text{Y}}_{\text{f}}}{{\text{Y}}_{\text{x}}}=-\left(\frac{G_{x}+j\text{ ω}C_{x}}{G_{f}+j\text{ ω}C_{f}}\right)=-k_{g}\left(\frac{\text{σ}+j\;{\text{ωε}}_{0}\text{ε}}{G_{f}+j\text{ ω}C_{f}}\right)$$

where *G_x_* and *G_f_* are the conductance, *C_x_* and *C_f_* are the capacitances of the impedances **Z_x_** and **Z_f_**, respectively.

In Equation (6), *C_x_* and *G_x_* are directly connected with the determination of the sensor σ and ε. This involves the measurement of two parameters, which may be: (i) magnitude and phase in a single frequency; (ii) real and imaginary parts of **V_0_** at a single frequency; or (iii) two amplitudes at different frequencies. These three possibilities are mathematically equal, but differ with respect to the complexity of the circuit [25,36]. This work proposes an electronic circuit for instrumentation that uses the third option to estimate σ and ε of the material located between the probe electrodes.

According to Equation (6), two thresholds can be identified by the ratio of *G_x_G_f_^−1^ and C_x_C_f_^−1^* when frequency *f* → 0 and *f* → ∞, according to Figure 2b. In practice, one should choose two frequencies located on each level. The voltage gain at lower frequency (A_0_) can be correlated with σ, and the voltage gain in the higher frequency (A_1_) with ε of the material located between the probe electrodes.

Silva [25] presented an approach to measure ε and σ of saline solutions using auto-balancing bridge circuit. However, these experiments were performed using signal generators for circuit excitation, and data was monitored and stored in the computer through an oscilloscope and a signal-processing algorithm. Solutions ε and σ were estimated by measuring the gain of the auto-balancing bridge circuit at two different frequencies.

**Figure 3:**
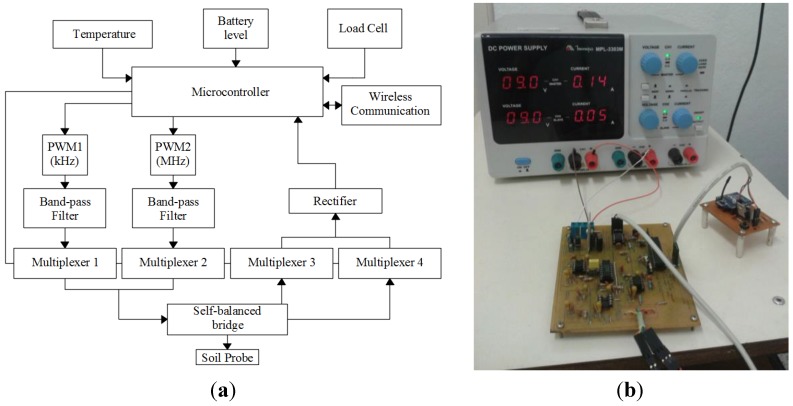
(**a**) Signal-conditioning unit circuit diagram; (**b**) Signal-conditioning unit implemented circuit.

The present work describes the development of an embedded system in order to perform all tasks proposed in Silva [25], and to measure the soil parameters ε and σ for agriculture purposes. Furthermore, the system measures temperature to evaluate its effect on the measurements of ε and σ. Figure 3 shows the circuit diagram and the signal-conditioning unit.

In the circuit diagram shown in Figure 3a, the microcontroller provides Pulse Width Modulation (PWM) signals at different frequencies (100 kHz and 5 MHz) with 50% working cycle. These signals pass through bandpass filters to make them closer to sine functions. Therefore, they can be used as the excitation source for self-balanced bridge circuit.

The input and output of self-balanced bridge circuit are measured by the analog-to-digital converter (ADC) of the microcontroller. However, these signals are rectified before performing the reading. The multiplexers are meant to direct the power signals to the auto-balancing bridge circuit and to select which signal is read by the ADC: input signals or output. In addition, the microcontroller performs measurements of the temperature sensor located within the probe circuit and battery power level. This data is sent wirelessly to the master program installed on a computer. The measurement of load cell signal is used only in the system calibration process, which will be described later.

It is possible to use a variety of microcontrollers and amplifiers to implement the proposed circuit. For this implementation, the auto-balancing bridge uses a 680 Ω resistor, a 270 pF capacitor and an AD8065 FastFET operational amplifier. The microcontroller is a PIC16F873A. The multiplexers employ a CMOS quad bilateral switch CD4066. Both band-pass filter and rectifier are implemented with a TLE2072 operational amplifier.

### 2.3. Calibration and Testing

#### 2.3.1. Calibration Using Salts Solutions

To perform the calibration procedure seven different substances were used as references, all of which having both σ e ε known, as follows: μ

1. Air $\text{(σ = 0 μS}\cdot {\text{cm}}^{-1}\text{; ε = 1)}$;
2. Deionized water. $\text{(σ = 4.5 μS}\cdot {\text{cm}}^{-1}\text{; ε = 80);}$
3. Drinking water. $\text{(σ = 68.7 μS}\cdot {\text{cm}}^{-1}\text{; ε = 80);}$
4. Solution of water and NaCl 1 $\text{(σ = 145.8 μS}\cdot {\text{cm}}^{-1}\text{; ε = 80);}$
5. Solution of water and NaCl 2 $\text{(σ = 348.4 μS}\cdot {\text{cm}}^{-1}\text{; ε = 80);}$
6. Solution of water and NaCl 3 $\text{(σ = 838.8 μS}\cdot {\text{cm}}^{-1}\text{; ε = 80);}$
7. Ethanol fuel $\text{(σ =7.9 μS}\cdot {\text{cm}}^{-1}\text{; ε = 24).}$

The constant σ of each material was measured using an apparatus with automatic temperature compensation. On the other hand, ε was obtained from the literature. These values were adopted as conventional real values, both for σ and ε of the solutions.

The tests were conducted in a climate chamber with temperature controlled at 25 ± 1 °C. Forty measurements were performed for each situation. So, there were seven treatments and 40 replicates for each of the three designed probes.

For the calibration models, circuit voltage signals from the gains A_0_ and A_1_ of the auto-balancing bridge at each frequency were respectively correlated with σ and ε using linear regression.

After obtaining these models, the effect of temperature was evaluated. For this purpose, measurements were taken under seven different temperature conditions described above. Subsequently, the cooling chamber was configured to vary the temperature from 5 °C to 45 °C with 5 °C steps, with each step lasting for 2.5 h. During this process, the supervisory software carried out data acquisition.

According to the solutions measurements of σ and ε accomplished at different temperatures, two correction models were obtained empirically. After, they were applied in the soil conductivity and water content measurements. Both of them have also being compared to the correction models proposed by Rhoades *et al.* [37] Equation (7) and Chanzy *et al.* [38] Equation (8). Root mean square error (RMSE) and coefficient of determination (*R*^2^) were employed to compare the corrections difference:

(7) $${\text{σ}}_{25}=\left[1-0.20346\left(\frac{T-25}{10}\right)+0.03822{\left(\frac{T-25}{10}\right)}^{2}-0.00555{\left(\frac{T-25}{10}\right)}^{3}\right]{\text{σ}}_{m}$$

where σ_25_ is the electrical conductivity adjusted to 25 °C, and σ_m_ is the conductivity measured according to the temperature *T*. For the complex relative permittivity, we have:

(8) $${\text{ε}}_{25}={\text{ε}}_{m}+\text{α}(25-T)$$

where α is obtained empirically, based on the soil θ and σ; here we adopt α = 0.114 [38]. This model will be employed and compared to the one proposed in this work.

#### 2.3.2. Tests Conducted with Soil

**Figure 4:**
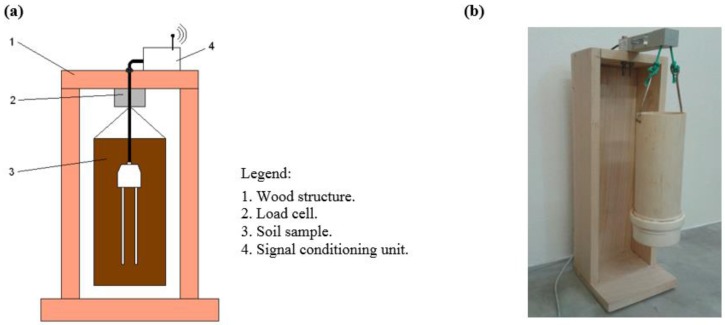
(**a**) Scheme support structure of the soil samples; (**b**) Structure of weighing the soil samples.

Two tests using clay and sandy soil were performed. These tests consisted of saturating a soil sample with water and submitting it to a drying procedure. Meanwhile, the sample mass and the signals corresponding to σ and ε were measured by the signal-conditioning unit and stored in the supervisory program database. A load cell suspending the sample measures its mass, as illustrated in Figure 4. The signal-conditioning unit is also responsible for performing these measurements.

The soil drying procedure consists on placing the structure shown in Figure 4 in the climate chamber, which was scheduled to reach 50 ± 1 °C for three hours and go back to 25 ± 1 °C for five hours, until the soil gets quite dry, and its weight practically stops changing. After this test, the soil moisture content by weight using a standard oven method for each measuring point is obtained. Finally, a correlation model between dielectric constant of the soil and the soil water content is fitted using linear regression.

Attempting to minimize the biasing influences on the sensor readings about the vertical gradient of water content from soil saturation to field capacity, the weighting container has restricted dimensions—75 mm diameter and 200 mm height.

The structure presented in Figure 4 was developed to find the calibration models for a specific soil according to a standard procedure. This procedure is necessary since the measuring circuit to obtain σ, ε and the soil temperature also detects the mass inside the weighing recipient. The acquired information is sent to the supervisory software that adjusts the calibration model. The novel calibration device greatly enhances the estimation precision of θ.

For experimental validation, we took 2608 measurements of sandy soil and 1780 measurements of clay soil, during a period of 12 and 16 days, respectively. The volumetric water content ranges for sandy and clay soil were 0.02–0.40 and 0.02–0.43. The volumetric soil water content was calculated multiplying the soil water content on weight basis by the soil bulk density ρ, which in this case is 1.27 g·cm^−3^ for sandy and 0.92 g·cm^−3^ for clay soils.

In order to validate the correlation models between the dielectric constant and the water content, we employed the K-fold cross validation method. This analysis consists on randomly dividing the original sample data in K subsamples. After that, a single subsample is retained to be used as the validation, and the remaining K − 1 subsamples are used as training data. The cross-validation process is then repeated K times (the folds), until each K subsample is employed once as the validation data.

The K results from the folds then can be averaged to produce a single estimation. The advantage of this method over repeated random sub-sampling is that all observations are used for both training and validation, and each observation is used for validation exactly once [39]. Here we applied K = 10 and 100 rounds.

The models proposed to predict θ based on ε were compared with models developed by Topp *et al.* [40], Ledieu *et al.* [41] and Malicki *et al.* [42]. These models are represented by Equations (9)–(11), respectively. To quantify the models accuracy, we analyzed the RMSE and *R*^2^ between observed and predicted values:

(9) $$\text{θ}=4.3\cdot 10^{-6}{\text{ε}}^{3}-0.00055{\text{ε}}^{2}+0.0292\text{ε}-0.053$$

(10) $$\text{θ}=\text{0.1264}\sqrt{\text{ε}}-\text{0.1933}$$

(11) $$\text{θ}=\frac{\left(\sqrt{\text{ε}}-0.819-0.168\text{ρ}-0.159{\text{ρ}}^{2}\right)}{7.17+1.18\text{ρ}}$$

## 3. Results and Discussion

### 3.1. Salt Solutions

**Figure 5:**
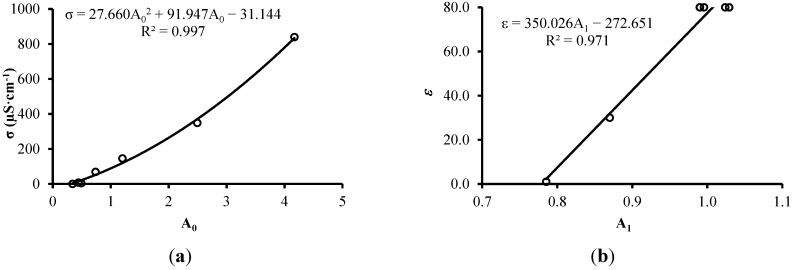
Relationships between σ-A_0_ (**a**) and ε-A_1_ (**b**).

Figure 5a shows the correlation between σ measured by a conductivimeter and A_0_ for a temperature of 25 ± 1 °C. Figure 5b shows the correlation between ε of the substances and A_1_ for a temperature of 25 ± 1 °C.

This experiment is based on work by Silva [25], which employs air (ε = 1, σ = 0), oil (ε = 2, σ = 0), isopropanol (ε = 19, σ = 0.06 μS·cm^−1^), glycol (ε = 37, σ = 3 μS·cm^−1^), deionized water (ε = 79, σ = 2 μS·cm^−1^) and water + salt (ε = 79, σ = 26 μS·cm^−1^) to demonstrated the first order relationship between the auto-balancing bridge circuit gain at high frequency and the dielectric constant of these substances. Assuming the same first order relationship and measuring principles, we use only three reference points of ε (Figure 5b).

**Figure 6:**
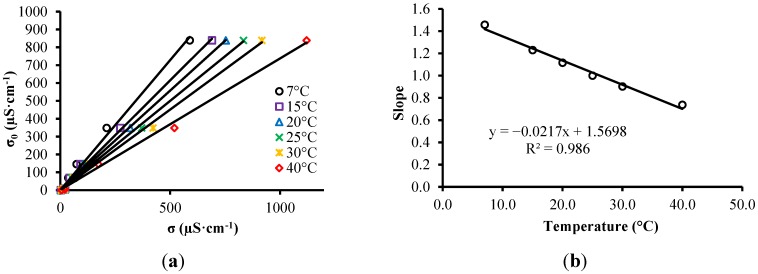
(**a**) σ-σ*_0_* relationship; (**b**) Correlation between temperature and slope of the fitted models for each temperature.

The effect of temperature on the measurement results of σ were evaluated as a function of the slopes of correlation equations between the values of electrical conductivity measured by the proposed system and electrical conductivity values measured by a conductivimeter (σ_0_) at different temperatures, as shown in Figure 6a. Thus, a model of the average temperature of each solution and the slope of each model was obtained, as shown in Figure 6b. With this model, it is possible to correct the measured σ and present the measurement result corrected for 25 °C, according to Equation (12):

(12) $$\sigma_{25}=(-0.0217T+1.5698)\sigma_{m}$$

**Figure 7:**
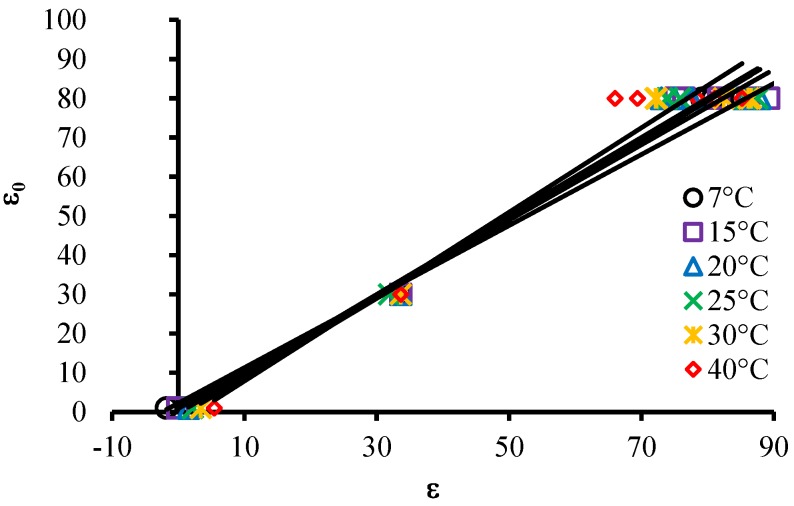
Models for comparison between measured values and true conventional values of ε for each different temperature.

To evaluate the effect of temperature on the measured results of ε the same procedure described above was adopted. The slopes of correlation patterns between the measured value of ε and the conventional true value of ε (ε*_0_*) were used for each temperature, as shown in Figure 7.

**Figure 8:**
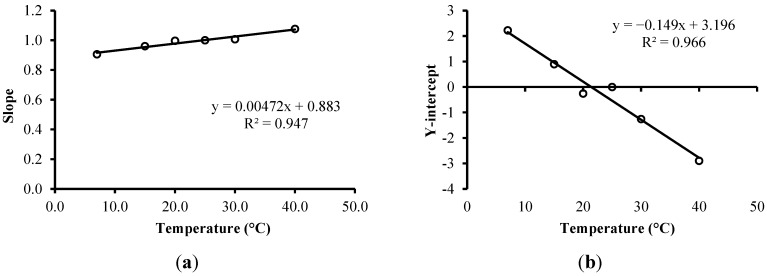
(**a**) Correlation between the temperature of the solutions and the slopes of the models for each temperature; (**b**) Correlation between the temperature of the solutions and the Y-intercepts of the models for each temperature.

Two models were fit between the average temperature of each solution and slopes and Y-intercepts, as shown in Figure 8. Thus, it is possible to correct the measured ε and present the measurement result at a reference temperature of 25 °C, according to Equation (13).

Equation (13) presents the correction model for ε empirically developed in this work based on the temperature:

(13) $${\text{ε}}_{25}=(0.00472T+0.8834){\text{ε}}_{m}-0.1494T+3.1957$$

where ε_25_ corresponds to ε adjusted to 25 °C, and ε_m_ is ε measured according to the temperature *T*.

### 3.2. Soils

**Figure 9:**
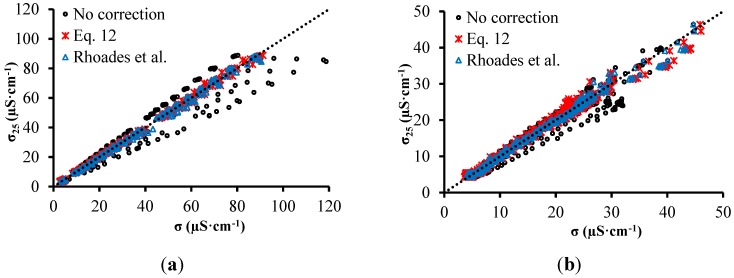
σ-σ_25_ relationship for sandy soil (**a**) and clay soil (**b**).

The measurement of σ is greatly influenced by temperature, as has already been observed in the tests with solutions. Figure 9 shows graphs of correlation between σ at 25 °C (σ_25_) and σ at all temperatures (20, 25, 30, 35 and 40 °C), measured before and after correction with temperature employing the correction model presented in this work (Equation (12)) and also the model proposed by Rhoades *et al.* [37] (Equation (7)). Table 1 shows *R*^2^ and RMSE, after and before the σ correction according to the temperature.

**Table 1:** Results from the comparison between σ correction models based on temperature.

	Sandy Soil		Clay Soil	
	RMSE	*R*^2^	RMSE	*R*^2^
**No correction**	6.7727	0.9321	1.9827	0.9598
**Equation 12**	0.9676	0.9986	1.0620	0.9886
**Rhoades *et al.***	1.3751	0.9971	0.7929	0.9936

Before correction, the adjusted models showed RMSE of approximately 6.8 μS·cm^−1^ for sandy soil and 2.0 μS·cm^−1^ for clay soil. Using the correction model presented by Rhoades *et al.* [37] Equation (7), it is possible to note that *R*^2^ increases while RMSE decreases, for both soils. Employing the model developed here, *R*^2^ increases while RMSE decreases even more, which indicates that the correction model proposed in this work is more accurated.

In this work we propose three prediction models: the first two Equations (14) and (15) are specific for sandy and clay soils; the third model was developed for both types of soil Equation (16). Even so, similar to Malicki *et al.* [42], it also takes into account ρ:

(14) $$\text{θ}=\text{21.98}\cdot {\text{10}}^{\text{-6}}{\text{ε}}^{3}-{\text{0.001419ε}}^{2}+\text{0.03638ε}-\text{0.1786}$$

(15) $$\text{θ}=\text{6.491}\cdot 10^{-6}{\text{ε}}^{3}-{\text{0.0004087ε}}^{2}+\text{0.01180ε}+\text{0.01907}$$

(16) $$\text{θ}=(4.518\cdot 10^{-6}{\text{ε}}^{3}-0.0002746{\text{ε}}^{2}+0.010984\text{ε}-0.006706)\text{ρ}$$

Equations (14) and (15) could be considered a recalibration of Topp’s equation, estimating water content based on ε through a third-degree polynomial, whose coefficient values change from soil to soil.

**Figure 10:**
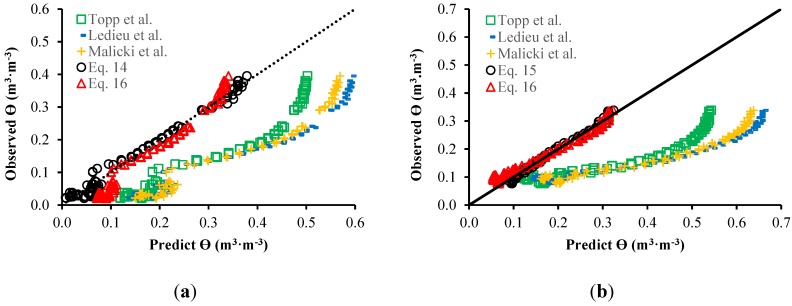
Relationship between predicted and observed θ for sandy (**a**) and clay soil (**b**).

Table 2 presents the results from the linear regression and cross-validation related to the models proposed in this work. Figure 10 illustrates the relation between θ predicted by each model and observed using the calibration system. Table 3 presents *R*^2^ and RMSE for each relation.

**Table 2:** Results of linear regression and cross-validation.

	Linear Regression	Cross-Validation	
	*R*^2^	*R*^2^	Standard Deviation
**Equation (14)**	0.9866	0.9856	0.0003
**Equation (15)**	0.9850	0.9839	0.0003
**Equation (16)**	0.9323	0.9298	0.0007

**Table 3:** Results from the comparison of models.

	Sandy Soil		Clay Soil	
	RMSE	*R*^2^	RMsSE	R^2^
**Topp *et al.***	0.05176	0.9319	0.02839	0.8798
**Lidieu *et al.***	0.04613	0.9542	0.2374	0.9191
**Malicki *et al.***	0.05114	0.9542	0.02334	0.9191
**Equation (14)**	0.01455	0.9866	-	-
**Equation (15)**	-	-	0.01001	0.9850
**Equation (16)**	0.03584	0.9804	0.02078	0.9797

According to Table 3, one can note that the prediction models for water content developed for each specific soil Equations (14) and (15) are more accurate due to the smaller RMSE and larger *R*^2^. Even so, the model represented by Equation (16), employed for both soils, also presented better precision than the regular models presented in the literature (Equations (9)–(11)).

**Figure 11:**
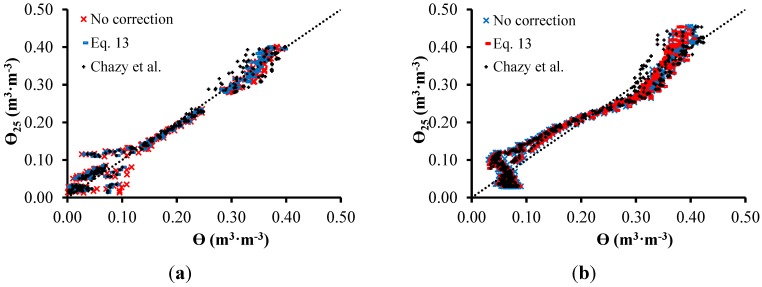
(**a**) θ-θ_25_ relationship for sandy soil (**a**) and clay soil (**b**).

Figure 11 shows graphs of correlation between ε at 25 °C (ε_25_) and ε at all temperatures, measured before and after correction with temperature employng the models proposed in this work Equation (13), and aslo the one presented by Chanzy *et al.* (Equation (8)). Table 4 ilustrates *R*^2^ and RMSE, after and before the correction of ε based on the temperature of the two soils.

According to Table 4, it is possible to observe that the results accuracy do not present significant changes due to the correction. The correction improves the results related to the sandy soil, but worsens the results related to the clay soil. Seyfried and Grant [43] mention a small and non linear effect for the temperature response, ranging from 5 °C to 45 °C, during the measurement of ε. According to the authors, this effect is small enough to be ignored for many applications.

**Table 4:** Results from the comparison between ε correction models based on temperature.

	Sandy Soil		Clay Soil	
	RMSE	*R*^2^	RMSE	*R*^2^
**No correction**	0.02322	0.9713	0.03232	0.9381
**Equation (9)**	0.02215	0.9761	0.03250	0.9380
**Chanzy *et al.***	0.02250	0.9704	0.03273	0.9367

Muñoz-Carpena [44] pointed out that the equipment that adopt the Time Domain Reflectometry (TDR), Amplitude Domain Reflectometry (ADR) and Frequency Domain Reflectometry (FDR) methods have a RMSE of 0.01 m^3^·m^−3^. The measurement system that uses the neutral probe is the most accurate, since it has a RMSE of 0.005 m^3^·m^−3^. Other inexpensive soil water content measurement systems presented RMSE of 0.02 to 0.03 m^3^·m^−3^ [12]. During the laboratory experiments, the measurement system developed in this work showed RMSE of 0.002 m^3^·m^−3^ for both clay and sandy soils, indicating good accuracy and cost-effectiveness with respect to traditional methods. Nevertheless, further experiments should validate the system accuracy under field conditions.

## 4. Conclusions

This paper presents the development, design and construction of a low-cost instrumentation system for measuring water content, apparent electrical conductivity and temperature of the soil. The measurement method is based on an auto-balancing bridge circuit. Experimental results obtained in the laboratory demonstrate the system accuracy, considered satisfactory for irrigation control. A comparison with other results in the literature also indicates the proposed system as a promising instrumentation device.

The proposed device corresponds to an efficient alternative to automatize irrigation systems, especially due to the satisfactory accuracy and low cost associated. The instrument was designed considering the final cost of the system to the farmer, which is a crucial concern while developing automation solutions for agriculture. Future work includes testing the prototype with slightly different and/or in undisturbed soils, and also manufacturing new devices for testing and field validation.
